# Pyrimidine nucleoside: inspiration for novel antimicrobial agent

**DOI:** 10.3389/fphar.2026.1773361

**Published:** 2026-03-05

**Authors:** Binjie Xu, Pengyu Li, Jiping Liu, Mingkai Li

**Affiliations:** 1 Department of Pharmacology, School of Pharmacy, Shaanxi University of Chinese Medicine, Xianyang, China; 2 Department of Pharmacology, School of Pharmacy, Air Force Medical University, Xi’an, China

**Keywords:** antibacterial agents, drug resistance, nucleosides, pyrimidine derivatives, structure-activity relationship

## Abstract

Antimicrobial resistance (AMR) is a worsening global health crisis, with drug repurposing emerging as a key mitigation strategy. Pyrimidine nucleosides are promising antibacterial scaffolds due to their easily modifiable structures and multi-therapeutic potential. However, related research faces challenges, including fragmented structure-activity relationships (SAR), unclear metabolism-efficacy correlations, and limited clinical translation strategies. This review categorizes these derivatives into cytosine and uracil/thymine analogs. It analyzes how lipidation, selenylation, and other structural modifications regulate antibacterial activity by modulating target binding, membrane permeability, and metabolic stability. Crucially, it elucidates their metabolic activation mechanism. As prodrugs, these derivatives require intracellular enzymatic phosphorylation to form active metabolites that inhibit nucleic acid synthesis, and their efficacy is dependent on intracellular enzyme levels and activity. Additionally, the review identifies core clinical translation barriers (host toxicity, narrow spectrum, insufficient AMR research) and proposes targeted optimization strategies (e.g., enzyme-guided modification and combination therapy). By integrating disparate structure-activity relationship and metabolic mechanism research, this work provides a novel systematic framework for developing pyrimidine nucleosides. Furthermore, it offers critical support to address the global antimicrobial resistance (AMR) crisis.

## Graphical Abstract

**Figure:**
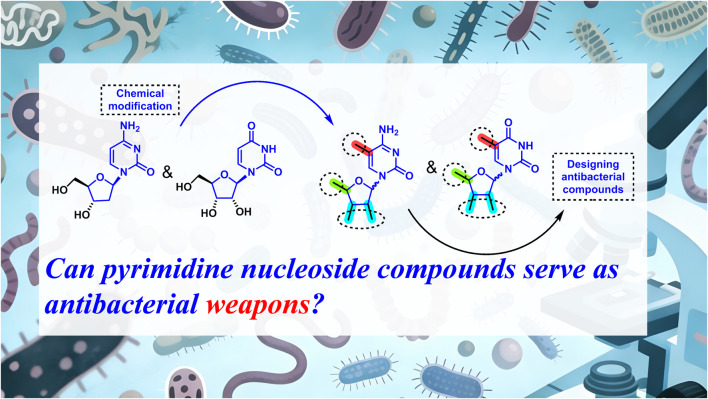

## 1 Introduction

Antimicrobial resistance (AMR) poses an increasingly global health challenge (Sovari et al., 2021; Wahid et al., 2024). Projections indicate that deaths attributable to drug-resistant bacterial infections will exceed 10 million by 2050 (Charani et al., 2022; Wagner et al., 2020). Unfortunately, the development of novel therapeutics lags far behind the emergence of resistant pathogens. This crisis necessitates that antimicrobial research transcend the limitations of traditional target saturation. Therefore, the focus must shift toward exploring compounds with novel mechanisms of action and chemical scaffolds. Current AMR response strategies primarily include: structural modification and optimization of existing drugs, application of molecular hybridization technology, development of chemical types targeting novel sites, and repurposing of marketed drugs (Annunziato, 2019; Bellapukonda et al., 2025). Drug repurposing has emerged as a critical supplementary strategy against AMR, owing to its shorter development cycle and well-established safety profile. Specifically, the repurposing of nucleosides has become a feasible approach to accelerate antimicrobial drug development (Dai et al., 2024). Due to their well-established pharmacokinetic profiles and safety data, pyrimidine nucleosides represent a highly promising research focus.

In the field of medicinal chemistry, heterocyclic compounds serve as essential building blocks for novel antimicrobial agents, among which pyrimidine nucleosides hold significant potential (Xu, 2020). These molecules possess a fundamental architecture where a ribose sugar is linked to a pyrimidine base via a glycosidic bond. Through precise modifications—such as nucleobase substitution, sugar moiety alteration, or linker engineering—their target binding affinity, antimicrobial activity, and membrane permeability can be significantly modulated (Hussein and Talib, 2024). Such structural modifications not only optimize pharmacokinetic profiles but also enable interference with bacterial metabolic pathways to overcome drug resistance, underscoring the critical role of structure-guided design in their development (Wagner et al., 2020; Xie et al., 2019).

Despite their demonstrated efficacy in disrupting nucleic acid synthesis and inhibiting key metabolic enzymes, current research faces significant hurdles. These include fragmented structure-activity relationships (SAR), a limited understanding of metabolic mechanisms, and a lack of viable strategies for clinical translation. To address these critical gaps, this review aims to achieve two primary objectives: first, to systematically categorize pyrimidine nucleosides into cytosine and uracil (thymine) analogues; second, to clarify the correlation between structural modifications and antibacterial activity while integrating metabolic pathway analysis to elucidate their anti-resistance mechanisms.

## 2 Structure-activity relationship and antibacterial activity of nucleoside pyrimidine derivatives

Pyrimidine derivatives constitute a privileged class of compounds with broad biological activities, exhibiting immense potential in modern drug design. Several clinically approved drugs, including fluorouridine (Krishna et al., 2024), cytarabine (Di Francia et al., 2021), sofosbuvir (Cholongitas and Papatheodoridis, 2014), capecitabine (Nandini et al., 2024), and molnupiravir (Masyeni et al., 2022; Richmond et al., 2024), belong to this category. Extensive research has demonstrated that these compounds, along with their fused derivatives, possess a diverse range of pharmacological properties, including anticancer (Wang R. et al., 2025), antiviral (Wang W. et al., 2025), anti-aging (Wei et al., 2025), and anti-inflammatory (Jacobson, 2025) therapies.

Notably, this scaffold has recently garnered substantial attention for its potent antimicrobial properties. The efficacy of these nucleosides is governed by the synergistic interplay between their nucleobase and sugar moieties: the former modulates binding specificity to bacterial targets, while the latter primarily regulates membrane permeability and metabolic stability.

### 2.1 Cytosine analogs

#### 2.1.1 Deoxyribocytidine derivatives (compounds 1–3)

**FIGURE 1:**
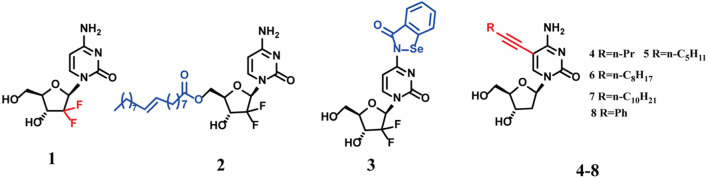
Chemical structures of compounds 1–8.

Chou et al. (1992) simplified the synthesis route proposed by Hertel et al. to obtain compound **1** (Gemcitabine) (Figure 1) (Hertel et al., 1988). Compound 1 (Gemcitabine) is a clinically approved deoxycytidine-based anticancer agent (Mai et al., 2023). It exerts antitumor effects by inhibiting ribonucleotide reductase, thereby reducing the supply of deoxynucleoside triphosphates (dNTPs) required for DNA synthesis in cancer cells (Clouser et al., 2011). Additionally, Gemcitabine acts as a broad-spectrum RNA virus inhibitor, interfering with both DNA and RNA synthesis (Shin et al., 2018). Notably, Sandrini et al. found that Gemcitabine induces bacterial death following activation by bacterial deoxyribonucleoside kinases, effectively killing *Staphylococcus aureus* and *Staphylococcus pyogenes* at concentrations below 0.002 μg/mL and 0.2 μg/mL, respectively (Sandrini et al., 2007). *In vitro* studies confirmed that Gemcitabine maintains potent inhibitory activity against methicillin-resistant *S. aureus* (MRSA), with MIC values comparable to those against methicillin-susceptible *S. aureus* (MSSA) strains (Table 1), indicating its potential to overcome drug resistance. However, it exhibits no significant activity against Gram-negative bacteria, including *E.coli* and *K. pneumonia* (Jordheim et al., 2012). The limited antibacterial spectrum is likely due to two factors. First, the lipopolysaccharide (LPS) barrier in Gram-negative bacteria hinders drug penetration. Second, the lower phosphorylation efficiency of deoxyribonucleoside kinase (dNK) in these bacteria leads to insufficient production of active metabolites (Sandrini et al., 2007).

**TABLE 1:** MICs of compounds 1–3 and 7.

Compounds	MIC (μg/mL)			
	MSSA	MRSA	GISA	*M.avium*
1	0.09	0.25	0.08	-
2	1.33	1.33	0.84	-
3	-	0.03–0.43	-	-
7	-	-	-	1–5

**FIGURE 2:**
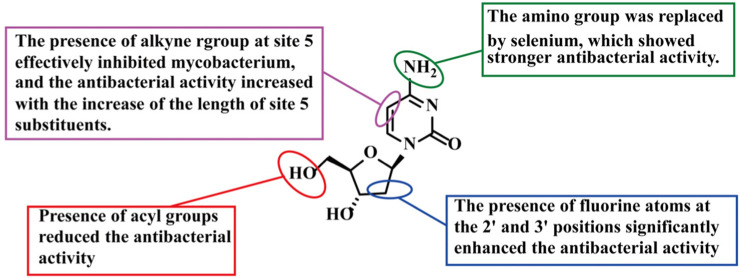
SAR of compounds 1–8.

Compound **2** (CP-4126) is a lipid-drug conjugate formed by combining compound **1** with elaidic acid (C18) at a 1:1 ratio. *In vivo*, it is hydrolyzed by esterases within plasma and tumor cells to release compound **1** (Venugopal et al., 2015). Similar to its parent drug, the released Gemcitabine requires metabolic activation. It must be phosphorylated intracellularly by bacterial deoxyribonucleoside kinases (dNKs) to form the active triphosphate metabolite, which then interferes with bacterial nucleic acid synthesis, thereby exert its antibacterial effects. Bergman et al. reported the synthesis of compound **2** in 2010 (Figure 1), noting that the introduction of the acyl group may influence its antibacterial activity (Bergman et al., 2011). While compound 2 displays marginally lower antibacterial activity than compound 1, it remains highly effective against MSSA, MRSA, and glycopeptide-intermediate *S. aureus* (GISA) strains (Table 1), indicating that resistance mechanisms do not compromise its efficacy (Jordheim et al., 2012). Compound 2 requires bacterial esterase-mediated conversion to compound 1 to exert antibacterial activity. In addition, its hydrophobic nature suggests that compound 2 enters bacteria via a different pathway compared with compound 1, which may account for its lower activity (Jordheim et al., 2012). But from SAR perspective, the introduction of an acyl group still represents a potential strategy to overcome the bottleneck of nucleosides against Gram-positive bacteria (Figure 2).

Compound **3** (Selenium-Gemcitabine) was synthesized through the covalent binding of the amino group of Gemcitabine with 1,2-benzisothiazol-3(2H)-one (Figure 1) (Chen et al., 2022). It exhibited potent activity against MDR MRSA (Table 1). Specifically, compound **3** exhibits stronger antibacterial a *t*-butyl ctivity than compound **1** against ATCC25923, MRSA-ZHF, and MRSA-XHX, but weaker activity against ATCC29213 and MRSA-CYZH (Chen et al., 2022). Mechanistic studies revealed that this selenium-containing derivative acts via a dual mechanism. Following metabolic activation, it inhibits DNA polymerase to interfere with nucleic acid synthesis and targets mitochondrial glutamate dehydrogenase (GDH) to disrupt energy metabolism. Although the reason for the variable activity across different strains requires further investigation, this modification strategy provides a new direction for the multi-target design of pyrimidine nucleosides. Collectively, SAR analysis reveals that replacing the amino group with selenium significantly enhances antibacterial activity. Furthermore, the presence of fluorine atoms at the 2′-position remains crucial for maintaining this potent activity (Figure 2).

#### 2.1.2 C-5 Propargyl-substituted cytosine derivatives (compounds 4–8)

Kumar et al. (Johar et al., 2005; Rai et al., 2005) first reported nucleosides with an alkyne substituent introduced at the cytosine C-5 position (compounds **4–8**) (Figure 1) and systematically evaluated their antimycobacterial activity against *Mycobacterium avium* (Alexandrova et al., 2022). The authors hypothesized that these compounds might act as inhibitors or competitive substrates in *Mycobacterium tuberculosis* enzyme-mediated nucleic acid synthesis. Following metabolic activation via phosphorylation, this action would interfere with bacterial DNA replication (Alexandrova et al., 2022). Among the synthesized compounds, the 5-substituted derivative **7** exhibited the strongest activity against *M.avium*, with potency comparable to that of the clinical drug rifampin (Table 1) (Johar et al., 2005). SAR analysis revealed a positive correlation between antibacterial activity and the length of the C-5 alkyl chain. Specifically, activity increased with increasing alkyl chain length (Figure 2) (Alexandrova et al., 2022).

### 2.2 Uracil (thymine) analogues

#### 2.2.1 Aliphatic/aromatic modified uracil derivatives (compounds 9–14)

**FIGURE 3:**
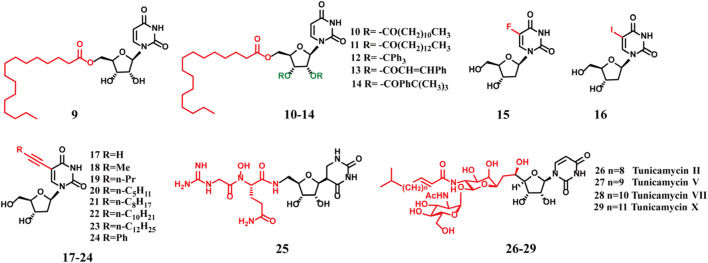
Chemical structures of compounds 9–29.

**FIGURE 4:**
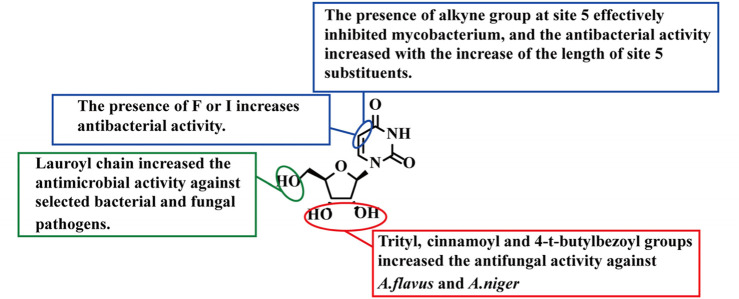
SAR of compounds 9–24.

In 2023, Munia et al. synthesized compounds **9–14** (Figure 3) as a new class of potential antibacterial analogs (Munia et al., 2023). These derivatives feature a uracil core modified with aliphatic chains of varying lengths and aromatic groups, aiming to explore how substituent hydrophobicity regulates antibacterial activity. The study evaluated five bacterial pathogens, including Gram-positive bacteria (*Staphylococcus aureus*, *Bacillus subtilis*) and Gram-negative bacteria (*Staphylococcus typhi*, *Prevotella aeruginosa*, *E. coli*). The results showed that compounds **11** and **12** exhibited significant inhibitory activity against *B. subtilis*, whereas compounds **9**, **10**, **13**, and **14** showed no activity against Gram-positive bacteria (Table 2). In addition, compound **11** showed good inhibitory activity against *S. typhi* (MBC = 16 μg/mL) and *E. coli* (MBC = 8 μg/mL), whereas compound **12** had relatively weaker antibacterial activity. Compounds **9** and **13** showed no inhibitory effect on all tested strains, while compounds **10**, **11**, and **14** maintained inhibitory effects on *P. aeruginosa* and *S. typhi* compared with previous studies (Munia et al., 2023). SAR analysis revealed that the introduction of a palmitoyl group at the uridine C-5′ and C-3′ positions of the inactive compound 9 was crucial for significantly enhancing its antibacterial activity (Munia et al., 2023). Furthermore, broader SAR studies indicated that the presence of lipophilic/acyl and aromatic substituents generally enhances antimicrobial or antifungal activity against selected pathogens (Ahad Hossain et al., 2024). However, it was also noted that excessive hydrophobic interactions between acyl chains can impair bacterial membrane permeability (Maher and Hassan, 2023). This reduces antibacterial efficacy by hindering the intracellular accumulation necessary for metabolic activation (Figure 4) (Judge et al., 2011).

**TABLE 2:** MIC of compounds 11–29.

Compounds	MIC (μg/mL)				
	*B. subtilis*	*S. suis*	*M. bovis*	*M. catarrhalis*	*Baccillus spp*
11	4	0.09	-	-	-
12	4	1.33	0.84	0.84	-
15	-	0.06–0.5	-	-	-
22	-	50	-	-	-
23	-	10	-	-	-
25	-	-	-	2	-
26	-	-	-	-	0.1–20
27	-	-	-	-	0.1–20
28	-	-	-	-	0.1–20
29	-	-	-	-	0.1–20

#### 2.2.2 Fluoropyrimidines and iodopyrimidine derivatives (compounds 15–24)

Compound **15** (Floxuridine, Figure 3) is a commonly used fluoropyrimidine antitumor drug in clinical practice (Myers et al., 1976; Proctor, 2008). Recent studies have found that it also has potential antibacterial activity (Li et al., 2022; Yeo et al., 2018). Li et al. (2022) confirmed that floxuridine exhibits potent *in vitro* activity against *Streptococcus suis* (Table 2), and *in vivo* experiments showed that it can significantly reduce the bacterial load in infected mice. Its antibacterial mechanisms are twofold: 1. inhibiting bacterial thymidylate synthase subsequent to activation by uridine kinase, and 2. disrupting the integrity of bacterial cytoplasmic membranes, which in turn downregulates the expression of virulence genes such as SLY (Li et al., 2022). This study confirmed that the antitumor toxicity of fluoropyrimidine compounds can be converted into antibacterial activity, providing a theoretical basis for the antibacterial reuse of antitumor nucleosides.

*Staphylococcus aureus* represents a major human disease-causing organism and is frequently recovered from the respiratory tract of individuals with cystic fibrosis (CF) (Stone and Saiman, 2007). Previously, the treatment for this bacterium has involved the use of sulfamethoxazole (SMX)/trimethoprim (TMP), either alone or in combination with rifampin (Stone and Saiman, 2007; Ellis and Lewis, 2005). Compound **16** (idoxuridine) (Figure 3) is an early-approved antiviral drug, and its antibacterial activity relies on a synergistic effect with TMP/SMX (Zander et al., 2010). Compound **16** acts as a thymidine analog via a dual mechanism. It competitively inhibits thymidine uptake, reducing the supply of natural precursors, and is simultaneously transported into the bacterial cell. Once inside, it undergoes metabolic activation via phosphorylation to disrupt nucleic acid synthesis; meanwhile, TMP/SMX inhibits dihydrofolate synthetase, blocking the *de novo* synthesis pathway of thymidine mediated by folate (Zander et al., 2010). This combination achieves dual blockade of salvage and *de novo* synthesis, significantly enhancing antibacterial activity. Empirical evidence demonstrates that co-administering idoxuridine with TMP/SMX reverses thymidine-induced resistance, thereby fully restoring efficacy against resistant *S. aureus* strains (Zander et al., 2010). Meanwhile, SAR analysis indicates that the introduction of fluorine or iodine substituents at the 5-position of the pyrimidine ring contributes to the enhancement of antibacterial activity (Figure 4).

In 2005, Kumar et al. (Johar et al., 2005; Rai et al., 2005) synthesized a series of pyrimidine nucleosides (**17–24**, Figure 3) by introducing an alkyne group at the C-5 position of 5-iodo-2′-deoxyuridine using the Sonogashira coupling reaction (Rai et al., 2007; Srivastav et al., 2007). *In vitro* activity tests showed that these derivatives inhibit the growth of mycobacteria, including clinically relevant *Mycobacterium tuberculosis* (Alexandrova et al., 2022), providing a potential nucleoside scaffold for the structural design of anti-mycobacterial drugs. Among these derivatives, compounds **22** and **23** exhibited superior antibacterial activity against *Mycobacterium bovis*, with compound **23** demonstrating significantly higher potency than compound **22** (Table 2). SAR analysis further reveals that extending the carbon chain at the 5-position of the pyrimidine base enhances antibacterial activity; generally, longer alkyl chains yield greater potency (Figure 4) (Alexandrova et al., 2022). This trend provides an important reference for the subsequent structural optimization of such compounds.

#### 2.2.3 Naturally occurring pyrimidine nucleosides (compounds 25–48)

Compound **25** (Pseudouridimycin) (Figure 3) is a natural nucleoside isolated from the fermentation broth of *Streptomyces* sp (Mosaei and Harbottle, 2019). Wang et al. synthesized this compound using 5′-amino pseudouridine and *N*-hydroxy dipeptides as starting materials (Wang X. K. et al., 2022). *In vitro* experiments demonstrated that pseudouridimycin exhibits potent antibacterial activity against both sensitive and resistant *Streptococci*, as well as against *M. catarrhalis* (Table 2) (Maffioli et al., 2019). SAR analysis identified the *N*-hydroxy group, glutamine residue, and guanidino group as key moieties for sustaining this antibacterial activity. Conversely, modification or deletion of any of these groups leads to a marked increase in the compound’s MIC against *Streptococci* and *M. catarrhalis* (Figure 4) (Maffioli et al., 2019). This suggests that natural pyrimidine nucleosides may have an inherent advantage in combating drug-resistant bacteria.

Compounds **26–29** (Figure 3) were isolated from the fermentation broth of *S.hemolyticus* or *Staphylococcus flavochromogenes* (Takatsuki et al., 1971). In 2017, Ichikawa et al. reported the total synthesis of Tunicamycin V (Yamamoto et al., 2018). Its chemical structure consists of three parts: *N*-acetylglucosamine, a fatty acyl side chain, and tunicaminyl-uracil (Bugg and Kerr, 2019). The antibacterial mechanism of these compounds involves inhibiting MraY and WecA, key enzymes in bacterial cell wall synthesis, thereby disrupting cell wall precursor synthesis and impairing cell wall integrity (Yamamoto et al., 2018; Bugg and Kerr, 2019). Antimicrobial activity tests showed that Tunicamycin exhibit significant inhibitory activity against Gram-positive bacteria such as *Bacillus* spp. (MIC = 0.1–20 μg/mL), *streptococci*, and *staphylococci*. SAR studies further confirmed that the tunicaminyl-uracil structural unit is essential for MraY inhibitory activity. Moreover, its spatial conformation directly affects the compound’s binding efficiency to the enzyme’s active pocket (Figure 4) (Yamamoto et al., 2019).

**FIGURE 5:**
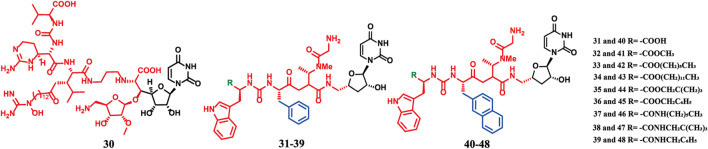
Chemical structures of compounds 30–48.

**FIGURE 6:**
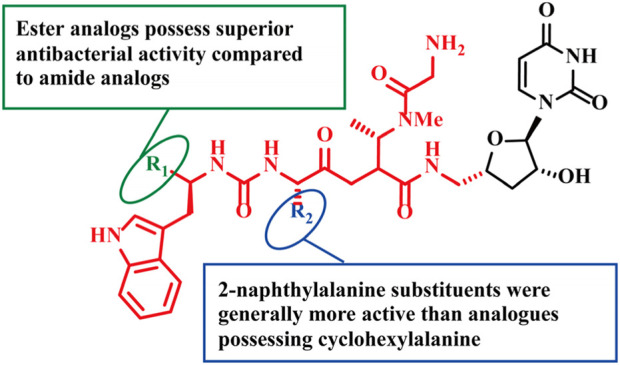
SAR of compounds 30–48.

Compound **30** (Muraymycin A1, Figure 5), a representative of the Muraymycin class, acts as a substrate analog to competitively inhibit the essential enzyme MraY. It exhibits a broad antibacterial spectrum covering both Gram-positive and specific Gram-negative bacteria. It demonstrates potent activity against *S. aureus* and specific *E. coli* strains, while also being effective against *Enterococci* (McDonald et al., 2002). However, this potency is severely compromised against wild-type *E. coli*, which is speculated to be due to the lower permeability of the outer membrane of wild-type *E.coli*, limiting the intracellular accumulation of the drug (McDonald et al., 2002). SAR demonstrated that fatty acid ester derivatives bearing charged groups exhibit significantly higher antibacterial activity than non-ester derivatives. Furthermore, activity was found to be positively correlated with the length of the fatty acid carbon chain (Figure 6) (McDonald et al., 2002). This finding provides a clear modification direction for improving the Gram-negative bacterial activity of Muraymycin class compounds.

Compounds **31–48** (Figure 5) are lipophilic amide-modified analogs synthesized based on the Sansanmycin family scaffold, with modification sites concentrated at the pseudo-peptide terminus of Sansanmycin (Tran et al., 2017). Similar to other nucleoside antibiotics, they function by inhibiting the essential enzyme MraY (Tran et al., 2017). This class of compounds exhibits activity restricted to *M.tuberculosis* H37Rv and no significant inhibition against common pathogens such as *S. aureus* and *Prevotella aeruginosa* (Table 3) (Tran et al., 2017). SAR revealed that analogs containing a 2-naphthyl proline substituent have higher activity than those with cyclohexyl proline substituents (Figure 6) (Tran et al., 2021). The reason is that 2-naphthyl has higher lipophilicity, which enhances the compound’s penetration into macrophages, thereby improving the inhibitory effect on intracellular *Mycobacterium tuberculosis* (Tran et al., 2017). This study provides a reference for the intracellular targeting design of anti-tuberculosis drugs.

**TABLE 3:** MICs of compounds 31–48.

Compounds	MIC (μg/mL) *Mtb*.H37 Rv	Compounds	MIC (μg/mL) *Mtb*.H37 Rv	Compounds	MIC (μg/mL) *Mtb*.H37 Rv
31	0.062 ± 0.015	37	90.1 ± 0.5	43	149.8 ± 9.3
32	0.028 ± 0.0015	38	0.0164 ± 0.0008	44	0.0164 ± 0.0002
33	0.0125 ± 0.0002	39	33.1 ± 0.9	45	0.0103 ± 0.0002
34	103.5 ± 12.5	40	0.148 ± 0.016	46	72.1 ± 1.4
35	0.0043 ± 0.0011	41	0.0487 ± 0.0011	47	0.0326 ± 0.0009
36	0.0062 ± 0.0011	42	0.0209 ± 0.0009	48	57.7 ± 1.9

#### 2.2.4 Thymidine kinase/thymidylate kinase targeted derivatives (compounds 49–51)

**FIGURE 7:**
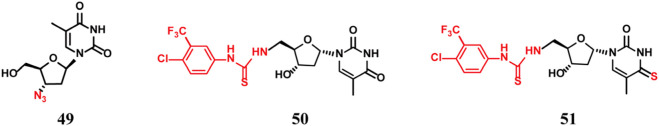
Chemical structures of compounds 49–51.

**FIGURE 8:**
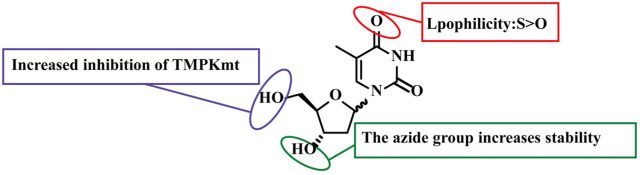
SAR of compounds 49–51.

Compound **49** (Zidovudine, Figure 7) is a clinically approved nucleoside reverse transcriptase inhibitor. Its antibacterial mechanism mirrors its antiviral mechanism. Upon phosphorylation by bacterial phosphokinases, it generates a triphosphate metabolite that incorporates into bacterial DNA. This metabolite then acts as a chain terminator to block DNA elongation (Antonello et al., 2021; Furman et al., 1986). *In vitro* antibacterial assays showed that compound **49** exhibits significant inhibitory activity against various Gram-negative bacteria, including *E. coli*, *Bacillus fragilis*, *Prevotella spp* (Table 4), while displaying no activity against Gram-positive bacteria (Ray et al., 2021). Further studies confirmed that the combination of compound **49** and tigecycline is effective in treating infections caused by carbapenem-resistant Enterobacteriaceae (Ray et al., 2021). SAR studies revealed that the introduction of uridine into compound **49** enhances its antibacterial activity against both Gram-negative and Gram-positive bacteria (Figure 8) (Levenfors et al., 2025). Its synergistic mechanism is the complementary pathways of DNA and protein synthesis inhibition, providing a new strategy for the treatment of multidrug-resistant Gram-negative bacterial infections.

**TABLE 4:** MICs of compounds 49–51.

Compounds	MIC (μg/mL)				
	*M. bovis*	*E. coli*	*Prevotella spp*	*B. fragilis*	*Mtb*
49	-	1–2	1–2	4–8	-
50	20	-	-	-	6.25
51	25	-	-	-	-

Thymidylate Kinase of *Mycobacterium tuberculosis* (TMPKmt) is a key enzyme in mycobacterial DNA synthesis, and it has only 22% amino acid sequence homology with human TMPK. This significant difference makes it a specific target for the development of anti-mycobacterial drugs. Daele et al. designed and synthesized 5′-thiourea-substituted α-thymidine derivatives, inspired by several active 3′-C-branched thiourea-substituted β-thymidine analogues. These derivatives show a similar relative orientation of the thymine base and arylthiourea moiety (Van Daele et al., 2007). As nucleosides, these compounds require metabolic activation via phosphorylation by upstream bacterial kinases to form their monophosphate derivatives, the active form that binds to TMPKmt as competitive substrates (Van Daele et al., 2007). Among them, compound **50** (Figure 7), as the first TMPK inhibitor, exhibited favorable inhibitory activity against *Mycobacterium bovis* and *M. tuberculosis* compared with its β-configured analogue (Table 4) (Van Daele et al., 2007). Compound **51** was synthesized starting from 3′,5′-O-diacetyl-α-D-thymidine, in which the oxygen atom at the 4-position of the thymine ring was replaced by sulfur. Interestingly, this modification increased TMPKmt inhibition by a factor of 3 compared with compound **50** (Figure 7) (Van Poecke et al., 2011). Furthermore, compound 51 exhibited 100% inhibition against M. bovis at a concentration of 25 μg/mL (Table 4) (Van Poecke et al., 2011). SAR analysis revealed that the isomer with the thymine base in a trans orientation relative to the aromatic substituent exhibited the most potent TMPKmt inhibitory activity. Notably, 4-thio modification of the pyrimidine base proved beneficial for the 5′-modified α-analogues, whereas it had the opposite effect on the 3′-modified β-analogues (Figure 8) (Van Poecke et al., 2011). This study confirmed TMPKmt as an effective target for pyrimidine nucleosides against *mycobacteria*, providing a clear direction for the design of specific anti-tuberculosis drugs.

### 2.3 The intrinsic correlation between pharmacophores and structure-activity relationships of pyrimidine nucleoside antimicrobials

The SAR of pyrimidine nucleoside antimicrobials follows a hierarchical logic based on three elements: a binary scaffold, site-specific modifications, and bacterial strain adaptation. The synergy of these three elements is central to their efficacy. A binary core scaffold is essential for antibacterial activity. This structure allows the compound to bind bacterial kinases and undergo phosphorylation. If the glycosidic bond is broken or the sugar ring is changed, the activity is lost (Sandrini et al., 2007). For example, modification of the sugar ring in Gemcitabine results in a dramatic loss of activity against MRSA (Sandrini et al., 2007; Johar et al., 2005; Author Anonymous, 2016).

With the integrity of the binary scaffold ensured, modifications to the base and sugar ring exhibit distinct functional divisions. Specifically, base modifications focus on optimizing target binding and antibacterial potency. Halogenation, alkynylation, and other modifications at the C-5 position enhance target affinity and membrane permeability (Johar et al., 2005). Furthermore, selenium/sulfur substitution at the C-4 position establishes a dual-targeting mode, significantly improving activity against multidrug-resistant (MDR) strains. In contrast, sugar ring modifications prioritize the improvement of membrane permeability (Bergman et al., 2011). Additionally, modifications with charged groups such as guanidine can further strengthen outer membrane penetration. This addresses the long-standing issue of insufficient activity of traditional analogs against Gram-negative bacteria (McDonald et al., 2002).

Notably, these modification strategies are not universal designs but require precise adaptation to bacterial strain characteristics. For example, Gram-positive bacteria prefer lipophilic base modifications to enhance target binding and intracellular transport, largely due to their simple cell wall structure (Sandrini et al., 2007; Jordheim et al., 2012); Gram-negative bacteria, constrained by the LPS barrier, must rely on dual modifications (charged groups and lipidation) of the sugar ring to achieve effective penetration (Bergman et al., 2011; McDonald et al., 2002); Mycobacteria, leveraging the species-specificity of their unique thymidylate kinase (TMPKmt), benefit from C-5 alkynylation and modifications targeting TMPKmt, enabling precise inhibition (Johar et al., 2005; Van Poecke et al., 2011). In summary, the binary scaffold provides the essential foundation for target binding. Complementing this, base and sugar ring modifications optimize efficacy and delivery, while bacterial strain adaptation ensures precise targeting.

## 3 Nucleoside metabolism is crucial for elucidating the antibacterial mechanisms of nucleosides

**FIGURE 9:**
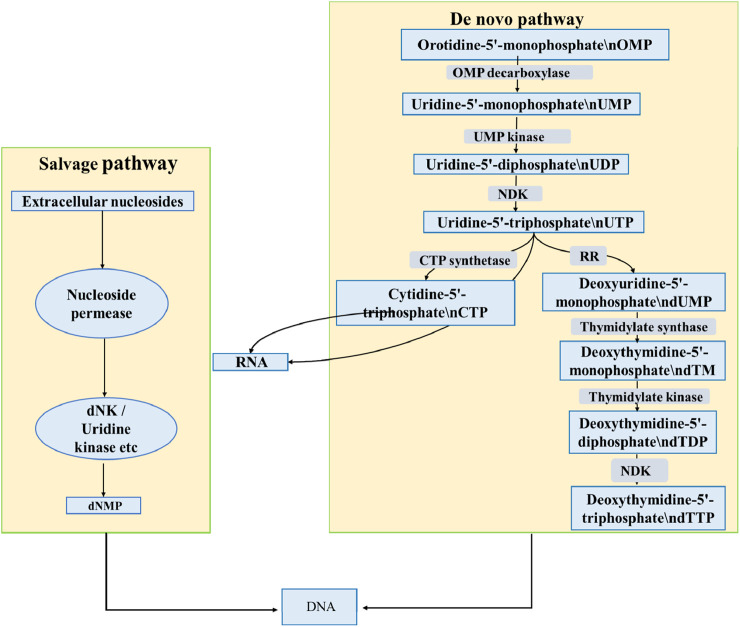
Pyrimidine nucleotide biosynthesis in bacteria via the *de novo* and salvage pathways.

Nucleosides are taken up and metabolized by cells via the same pathway as natural nucleosides (Sun and Wang, 2013), with their antibacterial activity relying heavily on bacterial-specific metabolic enzymes that activate the analogs into biologically active forms. Thus, understanding bacterial pyrimidine nucleotide synthesis, primarily mediated by *de novo* synthesis and salvage synthesis pathways, is essential to elucidating their antibacterial mechanisms (Figure 9):

*De Novo* Biosynthesis Pathway: Starting with orotidine-5′-monophosphate (OMP) as the initial substrate, it undergoes decarboxylation to generate uridine-5′-triphosphate (UTP) (Meanwell et al., 2020); UTP is further converted into cytidine 5′-triphosphate (CTP) under the catalysis of cytidine triphosphate synthetase (Jensen et al., 2008). The synthesis of deoxythymidine 5′-triphosphate (dTTP) requires two key reactions. Thymidylate synthase first catalyzes the methylation of dUMP to produce dTMP. Subsequently, this dTMP is phosphorylated by nucleoside kinase to generate dTTP (Costi et al., 2005; Vinogradov et al., 2006).

Salvage Synthesis Pathway: This pathway begins with the uptake of extracellular nucleosides via membrane transport proteins. The nucleosides are then converted into nucleotides through phosphorylation by enzymes like dNK and uridine kinase. These nucleotides are subsequently used for nucleic acid synthesis (Costi et al., 2005).

The antibacterial efficacy of pyrimidine nucleosides is inextricably linked to their metabolic activation within bacterial cells. These prodrugs must enter the cell and undergo sequential phosphorylation by kinases (e.g., dNK) to form active triphosphate metabolites (Ciccolini et al., 2016; Patel et al., 2024). For instance, gemcitabine relies on this pathway to generate dFdCTP, which acts as an RNA chain terminator, and dFdCDP, which inhibits ribonucleotide reductase (Yang et al., 2020). Consequently, the activity of bacterial metabolic enzymes and the stability of these phosphorylated products directly dictate the potency and spectrum of analogs such as gemcitabine and zidovudine.

## 4 Clinical translation challenges and research prospects of pyrimidine nucleoside antimicrobial agents

### 4.1 The core bottleneck of clinical translation

#### 4.1.1 The contradiction between target selectivity and host toxicity

A major hurdle in the clinical translation of pyrimidine nucleoside antimicrobials is host toxicity arising from the conservation of essential enzymes (Fàbrega et al., 2023; Kamzeeva et al., 2023). This is exemplified by Gemcitabine, which causes severe myelosuppression and pulmonary toxicity, thereby limiting its safety margin for antibacterial use (Chi et al., 2012). Consequently, toxicity remains a critical constraint for their development.

#### 4.1.2 Narrowing of antimicrobial spectrum and strain metabolic heterogeneity

A major limitation of existing pyrimidine nucleoside antimicrobials is their inability to penetrate the outer membrane of Gram-negative bacteria, resulting in a spectrum restricted primarily to Gram-positive strains. This is exemplified by Gemcitabine, which is highly active against *S. aureus* and *Mycobacterium avium*, but lacks efficacy against Gram-negative pathogens (Jordheim et al., 2012). Thus, overcoming this permeability barrier remains a critical challenge.

### 4.2 R&D optimization path

#### 4.2.1 Strategies for enhancing selectivity and permeability

Future structural modifications should focus on two key areas to maximize clinical potential. On the one hand, enhancing enzyme selectivity will minimize host toxicity. This can be achieved by leveraging the structural divergence between bacterial and human enzymes, as demonstrated in the design of compound **51** (Ray et al., 2021; Levenfors et al., 2025). On the other hand, optimizing membrane permeability through lipid conjugation strategies (e.g., CP-4126 analogs) will be essential to broaden efficacy against Gram-negative pathogen.

#### 4.2.2 Combination therapy based on pathway complementarity

Rational combination therapy is a highly promising strategy in anti-infective treatment. Combining antibiotics with different mechanisms can significantly improve antibacterial effects and delay the emergence and spread of drug-resistant strains. This provides an important approach to the growing problem of antimicrobial resistance (Liu et al., 2025).

For example, the combination of zidovudine and aminoglycosides shows synergistic inhibitory effects. Zidovudine is a nucleoside reverse transcriptase inhibitor. It mainly acts by interfering with nucleic acid synthesis. Aminoglycosides target bacterial ribosomes and inhibit protein synthesis. The two drugs act on different key steps in the bacterial life cycle. Their combination produces a synergistic effect and completely inhibits multidrug-resistant strains (Doléans-Jordheim et al., 2011). It effectively blocks bacterial growth and proliferation, offering a feasible solution for treating drug-resistant infections.

In addition, gemcitabine has some bactericidal activity against *Staphylococcus aureus*, but its effect is not long-lasting. Bacterial regrowth can be observed even at 256 × MIC. This indicates that monotherapy cannot fully eliminate pathogens and may easily induce resistance. To overcome this limitation, gemcitabine was combined with gentamicin. Synergistic bactericidal activity was achieved at the MIC of each drug (Jordheim et al., 2012). The combination rapidly and persistently inhibits bacterial growth. It also greatly reduces the frequency of resistance mutations and delays the emergence of resistant strains.

Therefore, developing rational combination regimens based on nucleosides is expected to become an important direction for clinical treatment of bacterial infections, especially those caused by drug-resistant strains. This approach has important theoretical value and clinical translation prospects.

## 5 Conclusion

Pyrimidine nucleosides with structural modifiability and metabolism-dependent activity serve as pivotal scaffolds for novel antimicrobial agents. This review summarizes the SAR of thymidine, uridine, and cytidine derivatives. It shows that specific nucleobase modifications optimize antibacterial activity by regulating binding, permeability, and stability. Integrating metabolic pathway analysis, we found that these compounds require intracellular activation within bacteria to work. This confirms the universal mechanism of metabolic activation. The mechanism is shared by anti-tumor, anti-viral, and anti-microbial nucleoside drugs (Ahmed, 2023). By analyzing the mechanism, we found a clear link between structural changes and metabolic efficiency. This offers a guide for designing new derivatives that target bacteria better and are less likely to develop resistance.

Unlike traditional studies that isolate SAR or metabolic mechanisms, this work establishes a clear correlation chain. This chain links structural modification, metabolic activation, and antibacterial activity, overcoming the limitations of fragmented existing research. This integrated perspective deciphers how lipidation, selenylation, and other modifications regulate antibacterial effects. It proposes a paradigm of “metabolism-oriented structural optimization”. Furthermore, it offers systematic solutions to clinical translation bottlenecks, such as a narrow antimicrobial spectrum and host toxicity (Hussein and Talib, 2024).

Current derivatives still face challenges of insufficient target specificity, a limited antimicrobial spectrum, and inadequate research on resistance mechanisms. Future studies should prioritize pathway-complementary combination therapy to broaden the antimicrobial spectrum and reduce resistance (Tran et al., 2017; Fàbrega et al., 2023), while leveraging the repurposing of clinically approved analogs (e.g., Gemcitabine) to accelerate clinical translation (Judge et al., 2011; Wang C. et al., 2022). With refined modification strategies and in-depth mechanism exploration, pyrimidine nucleosides are poised to become effective therapeutics for combating multidrug-resistant infections. In summary, this review highlights that metabolic activation serves as the critical mechanistic link between structural modification and antibacterial efficacy, offering a significant theoretical framework for the rational design of future nucleosides.
